# Celiac Artery Compression Syndrome Presenting As Recurrent Diabetic Ketoacidosis: A Diagnostic Challenge

**DOI:** 10.7759/cureus.17818

**Published:** 2021-09-08

**Authors:** Amr Salem, Ahmed Salem, Ameen Al-Aghil, Mohanad Soliman, Gerardo Carino

**Affiliations:** 1 Internal Medicine, Brown University, Providence, USA; 2 Internal Medicine, Alexandria Faculty of Medicine, Alexandria, EGY; 3 Critical Care Medicine, Brown University, Rhode Island, USA; 4 Pulmonary Critical Care, University of Arizona College of Medicine - Phoenix, Phoenix, USA; 5 Pulmonary and Critical Care, Brown University, Providence, USA

**Keywords:** celiac artery compression syndrome, cyclical vomiting syndrome, diabetic ketoacidosis, gastroparesis, median arcuate ligament syndrome

## Abstract

Celiac artery compression syndrome (CACS), also known as median arcuate ligament syndrome, can sometimes represent a diagnostic challenge. Here, we present the case of a 29-year-old man who presented with recurrent diabetic ketoacidosis (DKA), abdominal pain, and vomiting thought to be due to cyclical vomiting syndrome. However, the lack of a clear precipitant for DKA, the presence of chronic gastrointestinal symptoms, and a revealing physical examination of abdominal bruit led to clinical suspicion of CACS and its diagnosis after appropriate investigations. While angiography has traditionally been considered the gold standard diagnostic test, hemodynamic and geometric ultrasound criteria can, however, be diagnostic. The patient was managed by releasing the celiac artery through robotic surgery and serial monitoring as an outpatient revealed resolution of his symptoms and no further readmissions for DKA. This case highlights how a presumptive and erroneous diagnosis (cyclical vomiting syndrome) can misguide clinicians, especially when dealing with a rare diagnosis of exclusion.

## Introduction

Celiac artery compression syndrome (CACS), also known as median arcuate ligament syndrome, is a rare and easily missed cause of abdominal angina [1]. The median arcuate ligament passes superior to the origin of the celiac artery and represents a continuation of the posterior diaphragm. If it lies too low on the aorta, it can cause abdominal symptoms through compression of the celiac artery and/or celiac plexus. Here, we describe a case of recurrent abdominal pain secondary to CACS resulting in repeated episodes of diabetic ketoacidosis (DKA), allowing us to discuss the diagnosis and management of this rare but important condition.

## Case presentation

A 29-year-old man with a past medical history of poorly controlled type 1 diabetes mellitus with frequent admissions for DKA and a presumptive diagnosis of cyclical vomiting syndrome, presented with persistent abdominal pain, nausea, and vomiting. He reported 10 episodes of bilious vomiting over the two days prior to admission, and he had fallen several times. The pain, which was 7/10 in intensity, progressive, non-radiating, and epigastric, had worsened over the previous 18 months. The pain was mainly post-prandial and accompanied by vomiting. This had resulted in several hospital admissions, at which time the pain had been attributed to DKA. Despite being prescribed tramadol for rescue relief of breakthrough pain, the pain had become more difficult to manage. He was compliant with his medications, and there was no fever or symptoms suggestive of infection. The patient had also recently been started on desipramine and sumatriptan for cyclical vomiting syndrome. A previous gastric emptying study was inconclusive, and upper gastrointestinal endoscopy was negative. Avoidance of possible trigger foods for cyclical vomiting syndrome such as chocolate, cheese, and glutamate and dietary measures for gastroparesis in the setting of diabetes - including smaller, more frequent meals and avoidance of spices and fiber-rich foods - were unsuccessful.

In the emergency department, routine blood tests revealed sodium of 131 mEq/L, potassium 5.5 mmol/L, chloride 87 mEq/L, bicarbonate 15 mEq/L, anion gap 23, glucose 408 mg/dL, beta-hydroxybutyrate 5.35 mmol/L, arterial pH of 7.23, alkaline phosphatase 140 IU/L, lipase 13 U/L, aspartate aminotransferase 12 U/L, alanine aminotransferase 39 U/L, bilirubin 0.7 mg/dL, white blood cell count 7,400/µL, and hemoglobin of 13.7 g/dL. The patient satisfied the criteria for DKA, so he was started on IV insulin. His abdominal pain was attributed to a combination of DKA and cyclical vomiting syndrome. Physical examination was negative except for mild epigastric tenderness and an abdominal bruit.

**Figure 1:**
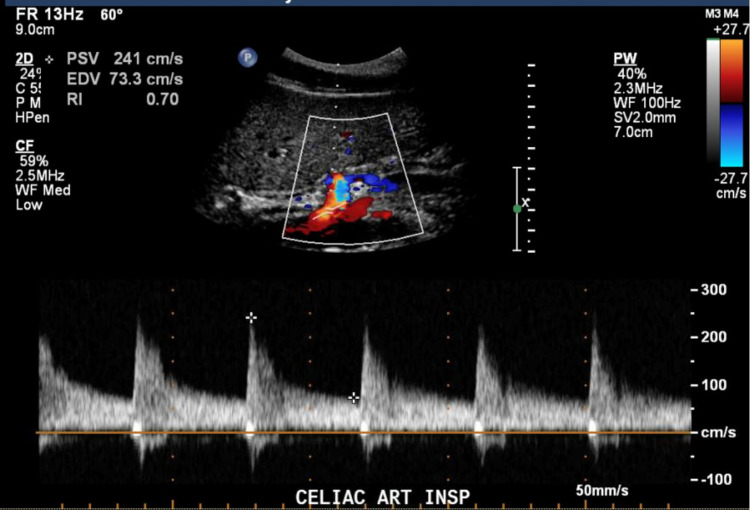
Celiac artery PSV at 241 cm/s during deep inspiration. PSV: Peak Systolic Velocity cm/s: Centimeters per second

**Figure 2:**
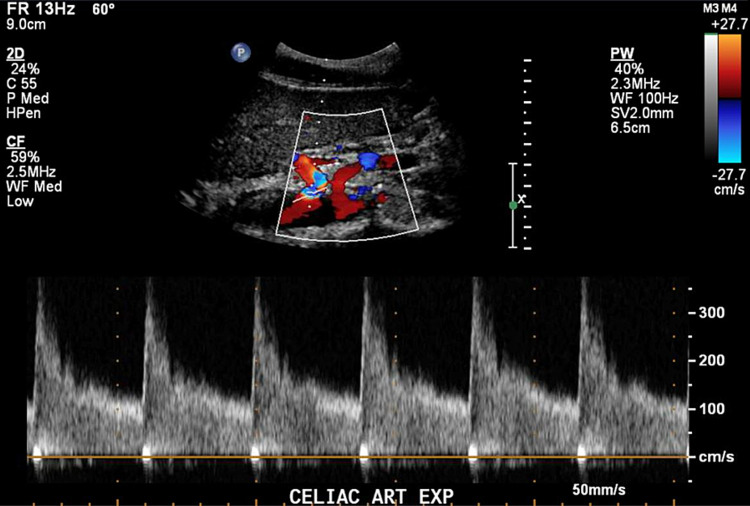
Celiac artery PSV at 367 cm/s during deep expiration. PSV: Peak Systolic Velocity cm/s: Centimeters per second

Computed tomography (CT) of the abdomen showed normal vasculature, no evidence of mesenteric ischemia, and moderate constipation. However, mesenteric duplex scanning showed evidence of significant celiac axis compression and elevated velocities in the proximal celiac artery (peak systolic velocity [PSV] 241 cm/s and end-diastolic velocity (EDV) of 73.3 cm/s on deep inspiration; PSV 367 cm/s and EDV 133 cm/s on deep expiration; Figures 1, 2).

He subsequently underwent robot-assisted release of the median arcuate ligament and dissection of the celiac plexus. He was seen in the outpatient clinic eight weeks later, where he reported significant improvements in his post-prandial pain.

## Discussion

Here, we present a diagnostically challenging case of recurrent abdominal pain initially attributed to DKA and cyclical vomiting. The patient had presented with recurrent DKA and metabolic derangements over 18 months despite reported compliance with his high-intensity insulin regimen. There had been no clear precipitant of his DKA, either in his history or in extensive investigations. The differential diagnosis included cyclical vomiting syndrome, gastroesophageal reflux disease, hiatus hernia, irritable bowel syndrome, chronic intestinal infections, and vascular and psychogenic causes.

The patient had previously received extensive investigations for abdominal pain, which excluded many of the differential diagnoses and leading to a presumptive diagnosis of cyclical vomiting syndrome. However, on this presentation, CT angiography (CTA) of the abdomen and pelvis was undertaken to explore the possibility of a vascular etiology in the setting of an epigastric bruit. He finally received a diagnosis of CACS based on Doppler hemodynamic criteria. In retrospect, the patient should not have received a diagnosis of cyclical vomiting syndrome as he had red flag signs including weight loss and metabolic derangements. While DKA could explain the patient's presentation, the persistence of symptoms between DKA episodes and a lack of precipitants of DKA should have prompted further investigations.

CACS is a rare syndrome with an incidence of two per 100,000 of the population, and it is more common in women aged 30-50 years [1]. As seen in our case, common symptoms of CACS include post-prandial epigastric or retrosternal pain, vomiting, nausea, decreased appetite, and weight loss [2]. More advanced cases can present with exercise-related abdominal pain that can be explained by the steal phenomenon, where blood is diverted from the superior mesenteric artery through collateral vessels to supply the celiac artery distribution [2]. Similar to our case, the presence of a significant, mainly expiratory murmur on examination along with post-prandial upper abdominal pain and weight loss should alert the clinician to the possibility of CACS [3,4].

Two theories have been proposed to explain the syndrome. First, the vascular theory suggests that compression of the celiac artery causes direct foregut ischemia or a post-prandial steal phenomenon. The second is neurogenic stimulation resulting in pain from celiac plexus stimulation, with resulting splanchnic vasoconstriction [5].

Thorough work-up for an alternative diagnosis is essential before diagnosing CACS and might include endoscopy, abdominal imaging, and functional studies. It is important to note that the finding of celiac artery compression on imaging is not diagnostic of CACS, which must be regarded as a clinical syndrome rather than an imaging finding [6]. Catheter angiography has traditionally been used to confirm CACS, but optimal views are necessary. Therefore, multi-slice and digital subtraction CTA have been suggested as alternatives to invasive angiograms [5]. However, for detection and follow-up, both CT and Doppler imaging have equal sensitivity and specificity [7].

Doppler interrogation of the celiac artery can be challenging due to limited acoustic access through obesity or an inability to obtain an adequate angle of incidence [8]. Gruber et al. [9] suggested assessment of flow changes in the celiac trunk and functional geometric changes such as celiac truck deflection as first-line screening for CACS. Using a peak systolic expiratory flow velocity of 350 cm/s and a celiac trunk deflection angle greater than 50° as diagnostic criteria, they reported a sensitivity of 83% and a specificity of 100% in a small cohort of volunteers [9]. A ratio of the PSV in the celiac artery at expiration compared to PSV in the abdominal aorta just below the diaphragm greater than 3:1 is another diagnostic criterion for CACS. In our case, the hemodynamic criteria were fulfilled [9], since he had a celiac artery peak expiratory systolic velocity of 367 cm/s, and a ratio between the celiac expiratory PSV and proximal aorta of 3.19 (367/115 cm/s).

Dynamic CTA of the abdomen and pelvis during inspiration and end-expiratory phase can help demonstrate dynamic compression of the celiac artery [10]. 3D contrast-enhanced ultrasonography is also a promising approach for non-invasive diagnosis [11]. Occasionally, the diagnosis of CACS is possible by EUS; for instance, when there is a low insertion of the median arcuate or high take-off of the celiac trunk [12].

Dietary changes are sometimes appropriate conservative management, although in our case, this was not successful [13]. Other management possibilities include decompression through surgical division of the median arcuate ligament along with celiac ganglion neurolysis, decompression and celiac artery reconstruction, and celiac artery stenting [14]. Several studies have shown that interventional endovascular therapy is less successful than external surgical approaches, probably because CACS is secondary to external compression of the celiac artery in contrast to other mesenteric occlusive diseases [15]. In our case, a robot-assisted technique was preferred to avoid the limitations of video endoscopy [15]. After surgical intervention, clinical improvements have been reported in 65% to 80% of CACS patients [16], and, as there is a recurrence risk after surgery, patients should receive serial follow-up [4].

Our patient was followed up as an outpatient at 8, 12, and 16 weeks, where he reported resolution of his post-prandial symptoms, and he had no further admissions for DKA. The resolution of these symptoms and the lack of further episodes of DKA confirmed that CACS was the correct diagnosis.

## Conclusions

In conclusion, recurrent DKA with no explainable cause requires comprehensive investigation. The diagnosis of CACS requires a high index of suspicion and can represent a diagnostic challenge, with dynamic imaging with hemodynamic measurements useful for diagnosis. About 75% of patients have a favorable prognosis after the surgical division of the median arcuate ligament, although careful selection for surgery is essential to ensure positive outcomes. Patients require serial follow-up to confirm that there has been an improvement in, and ideally resolution of, symptoms.

## References

[1] Paleti S, Boppanna V, Sobani ZA, McCarthy D, Rustagi T (2020). Compression regression: a rare but curable cause of postprandial abdominal pain. Dig Dis Sci.

[2] Karavelioğlu Y, Kalçık M, Sarak T (2015). Dunbar syndrome as an unusual cause of exercise-induced retrosternal pain. Turk Kardiyol Dern Ars.

[3] Koç M, Artaş H, Serhatlıoğlu S (2018). The investigation of incidence and multidetector computed tomography findings of median arcuate ligament syndrome. Turk J Med Sci.

[4] Marino RV, Lee RC (2016). Celiac artery compression syndrome and the vanishing bruit. Clin Pediatr (Phila).

[5] Karahan OI, Kahriman G, Yikilmaz A, Ok E (2007). Celiac artery compression syndrome: diagnosis with multi-slice CT. Diag Intervent Radiol.

[6] Rodriguez JH (2021). Median arcuate ligament syndrome: a clinical dilemma. Cleve Clin J Med.

[7] Bagley J, Stamile E, Berry JL, DiGiacinto D (2015). Case report: median arcuate ligament syndrome diagnosed with computed tomography and Doppler ultrasonography. Radiol Technol.

[8] Erden A, Yurdakul M, Cumhur T (1999). Marked increase in flow velocities during deep expiration: a duplex Doppler sign of celiac artery compression syndrome. Cardiovasc Intervent Radiol.

[9] Gruber H, Loizides A, Peer S, Gruber I (2012). Ultrasound of the median arcuate ligament syndrome: a new approach to diagnosis. Med Ultrason.

[10] Lakhani DA, Balar AB, Tarabishy AR (2021). Atypical cause of episodic abdominal pain and unintentional weight loss. Eur J Intern Med.

[11] Wang XM, Hua XP, Zheng GL (20181). Celiac artery compression syndrome evaluated with 3-D contrast-enhanced ultrasonography: a new approach. Ultrasound Med Biol.

[12] Ghevariya V, Bansal R, Sidhu L, Walfish A (20141). Celiac artery compression: EUS evaluation. Gastrointest Endo.

[13] Yoshida K, Kita K, Yamashiro S (2017). A 56-year-old female with celiac artery compression syndrome recovering through dietary changes and weight gain. J Gen Fam Med.

[14] Kohn GP, Bitar RS, Farber MA, Marston WA, Overby DW, Farrell TM (2011). Treatment options and outcomes for celiac artery compression syndrome. Surg Innov.

[15] Meyer M, Gharagozloo F, Nguyen D, Tempesta B, Strother E, Margolis M (2012). Robotic‐assisted treatment of celiac artery compression syndrome: report of a case and review of the literature. International J Med Robo Comp Assist Surg.

[16] Matsuura H, Okita A, Suganami Y (2020). Intermittent severe epigastric pain and abdominal bruit varying with respiration. Gastroenterology.

